# The Double-Stranded DNA Virosphere as a Modular Hierarchical Network of Gene Sharing

**DOI:** 10.1128/mBio.00978-16

**Published:** 2016-08-02

**Authors:** Jaime Iranzo, Mart Krupovic, Eugene V. Koonin

**Affiliations:** a National Center for Biotechnology Information, National Library of Medicine, Bethesda, Maryland, USA; b Institut Pasteur, Unité Biologie Moléculaire du Gène chez les Extrêmophiles, Paris, France

## Abstract

Virus genomes are prone to extensive gene loss, gain, and exchange and share no universal genes. Therefore, in a broad-scale study of virus evolution, gene and genome network analyses can complement traditional phylogenetics. We performed an exhaustive comparative analysis of the genomes of double-stranded DNA (dsDNA) viruses by using the bipartite network approach and found a robust hierarchical modularity in the dsDNA virosphere. Bipartite networks consist of two classes of nodes, with nodes in one class, in this case genomes, being connected via nodes of the second class, in this case genes. Such a network can be partitioned into modules that combine nodes from both classes. The bipartite network of dsDNA viruses includes 19 modules that form 5 major and 3 minor supermodules. Of these modules, 11 include tailed bacteriophages, reflecting the diversity of this largest group of viruses. The module analysis quantitatively validates and refines previously proposed nontrivial evolutionary relationships. An expansive supermodule combines the large and giant viruses of the putative order “Megavirales” with diverse moderate-sized viruses and related mobile elements. All viruses in this supermodule share a distinct morphogenetic tool kit with a double jelly roll major capsid protein. Herpesviruses and tailed bacteriophages comprise another supermodule, held together by a distinct set of morphogenetic proteins centered on the HK97-like major capsid protein. Together, these two supermodules cover the great majority of currently known dsDNA viruses. We formally identify a set of 14 viral hallmark genes that comprise the hubs of the network and account for most of the intermodule connections.

## IMPORTANCE

Viruses and related mobile genetic elements are the dominant biological entities on earth, but their evolution is not sufficiently understood and their classification is not adequately developed. The key reason is the characteristic high rate of virus evolution that involves not only sequence change but also extensive gene loss, gain, and exchange. Therefore, in the study of virus evolution on a large scale, traditional phylogenetic approaches have limited applicability and have to be complemented by gene and genome network analyses. We applied state-of-the art methods of such analysis to reveal robust hierarchical modularity in the genomes of double-stranded DNA viruses. Some of the identified modules combine highly diverse viruses infecting bacteria, archaea, and eukaryotes, in support of previous hypotheses on direct evolutionary relationships between viruses from the three domains of cellular life. We formally identify a set of 14 viral hallmark genes that hold together the genomic network.

## INTRODUCTION

A major discovery of environmental genomics and viromics over the last decade is that the most common and abundant biological entities on earth are viruses, in particular bacteriophages (1–5). In marine, soil, and animal-associated environments, virus particles consistently outnumber cells by 1 to 2 orders of magnitude. Viruses are major ecological and even geochemical agents that in large part shape such processes as energy conversion in the biosphere and sediment formation in water bodies by killing off populations of abundant, ecologically important organisms, such as cyanobacteria or eukaryotic algae (3, 5, 6). With the possible exception of some intracellular parasitic bacteria with highly degraded genomes, viruses and/or other selfish elements, such as transposons and plasmids, parasitize all cellular organisms. Complementary to their physical dominance in the biosphere, viruses collectively appear to encompass the bulk of the genetic diversity on Earth (7–9). The ubiquity of viruses in the extant biosphere and the results of theoretical modeling indicating that emergence of selfish genetic elements is intrinsic to any evolving system of replicators (10–13) jointly imply that virus-host coevolution has been the mode of the evolution of life ever since its origin (14–16).

Viruses and related mobile genetic elements (MGE) clearly have not evolved from a single common ancestor: indeed, not a single gene is conserved across the entire “greater virus world” (also known as the virosphere; here, the two terms are used interchangeably) or even in the majority of selfish elements (17, 18). However, different parts of the virosphere form dense evolutionary networks in which genomes of various selfish elements are linked through different shared genes (19–21). This type of evolutionary relationship results from extensive exchange of genes and gene modules, in some cases between widely different elements, as well as parallel capture of homologous genes from the hosts. Viruses with large genomes possess numerous genes that were acquired from the hosts at different stages of evolution; such genes are typically restricted in their spread to a narrow group of viruses. In contrast, the broader connectivity of the evolutionary network in the virus world derives from a small group of genes that have been termed virus hallmark genes, which encode key proteins involved in genome replication and virion formation and are shared by overlapping sets of diverse viruses (17–19). Virus hallmark genes have no obvious ancestors in cellular life forms, suggesting that virus-like elements evolved at a precellular stage of the evolution of life. The hallmark genes comprise only a small subset within the set of virus core genes, which for the purpose of this work will be defined as the genes that tend to have been retained in groups of related genomes along the course of evolution. The concept of core genes, which departs from the intuitive idea of genes with universal presence, accounts for the multiple evolutionary histories of distinct groups of viruses. The set of core genes also includes signature genes, i.e., genes that are highly prevalent within and specific to one particular group of viruses.

Due to the patchy gene distribution across the diversity of viruses and related MGE, standard methods of phylogenetics and phylogenomics have limited applicability in the study of the evolution of the virosphere outside relatively narrow, tight groups. Instead, methods for direct analysis of evolutionary networks are called for. These approaches benefit from the vast arsenal of mathematical concepts and tools that have been developed in different areas of network research (22–26). Recent application of network analysis methods to the comparative analysis of microbial and bacteriophage genomes has been productive, in particular, for the identification of preferred routes and patterns of horizontal gene transfer (HGT) (27–29).

The viromes and mobilomes (i.e., the supersets of viruses and other selfish elements) of the three domains of cellular life (bacteria, archaea, and eukaryotes) are fundamentally different. Although several families of double-stranded DNA (dsDNA) viruses are represented in both bacteria and archaea, no viruses are known to be shared by eukaryotes with any of the other two cellular domains, even at the family or order level (30). In bacteria and archaea, the virosphere is heavily dominated by dsDNA viruses, with relatively limited representation of single-stranded DNA (ssDNA) viruses and only a few narrow groups of RNA viruses. The eukaryotic part of the virosphere is sharply different, with a dominant presence of RNA viruses; however, dsDNA viruses of eukaryotes are also common and diverse (31). Altogether, the dsDNA viruses occupy most of the virosphere and include the largest viral genomes, thus presenting ample material for the construction of gene-sharing networks.

Bipartite networks, also known as 2-mode networks in the social sciences literature (32), are a natural way to represent, in the form of networks, complex systems that consist of two distinct classes of components (nodes). Straightforward examples from the field of biology are metabolic networks, composed of metabolites and enzymes, or pollination networks, composed of plants and their pollinators. Similarly, the network of gene sharing among viruses (due to common ancestry or HGT) calls for a bipartite network representation, where the two classes of nodes are viral genomes and homologous gene families. Historically, the lack of analytical tools to deal with bipartite networks has forced researchers to turn to monopartite projections (simplified versions of the network involving only one class of nodes), an approach that is not free from some degree of arbitrariness and can introduce biases (33). However, recent advances in network theory, in particular those related to module detection, allow for direct analysis of bipartite networks with minimal loss of information and simultaneous characterization of both classes of nodes (34).

Here we present a bipartite network analysis of all currently recognized families of dsDNA viruses and some related mobile genetic elements (MGE). Dissection of this network using formal analytical tools reveals extensive modularity of this part of the virus world, objectively identifies the set of viral hallmark genes, and quantitatively vindicates previously proposed scenarios for the origin of diverse groups of viruses.

## RESULTS

### Bipartite network of dsDNA viruses.

In order to develop a network representation of the relationships between all major groups of dsDNA viruses, we first had to identify the families of homologs that would become the nodes of the “gene family” class. To that end, we retrieved all predicted protein-coding sequences from more than 1,440 viral genomes and related MGE and grouped them into families by sequence similarity. Because of the large number and high diversity of the viral sequences, a multistep approach to the construction of these families was adopted. First, we developed an automated pipeline that combined sequence similarity analyses and algorithms for community detection in networks (see Materials and Methods). A comparison of the gene families obtained through this pipeline and the available clusters of orthologous genes for bacteriophages (POGs) (9, 35) and large nucleo-cytoplasmic DNA viruses of eukaryotes (NCVOGs) (36, 37) yielded a recall (average fraction of sequences in a POG or NCVOG grouped together in our analysis) of 0.92, and purity (1 minus the average fraction of false positives) of 0.89. In a subsequent step, some of the major families were manually curated to account for distant homology relations that, despite being strongly supported by previous reports, escaped automatic detection (see Materials and Methods for details).

We represented the web of relations among dsDNA viruses by means of a bipartite network in which the nodes belong to two classes: genomes and gene families. Edges connect a genome to every gene family that it contains; conversely, a gene family is linked to all genomes in which it is present. Compared to other network approaches based on genome-genome similarity metrics, the bipartite representation provides for explicit identification not only of clusters of genomes sharing gene sets but also of the genes that glue these genomes together.

**FIG 1:**
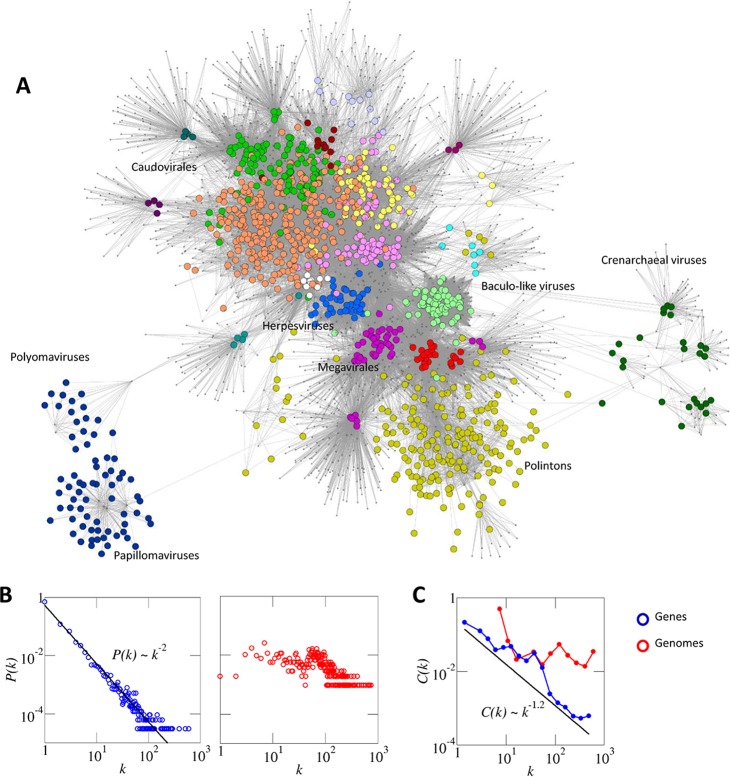
The dsDNA virus world as a bipartite network. Nodes corresponding to genomes are depicted as larger circles, and nodes corresponding to core gene families are depicted as dots. An edge is drawn whenever a genome harbors a representative of a core gene family. (A) The modular structure of the network is highlighted by coloring genome nodes according to the module to which they belong (color coding is as described for Fig. 4 to 6). The location of some major viral groups is indicated for illustrative purposes. (B) The degree distributions of genes (left) and genomes (right). In the case of genes, the best fit to a power law distribution is also shown. (C) The scaling of the clustering coefficient, *C*(*k*), with respect to the degree *k* (genes and genomes) suggests a hierarchical modular structure organized around high-level hallmark genes [large *k* and small *C*(*k*)] and low-level signature genes [small *k* and large *C*(*k*)].

Due to the high diversity of viral genomes, the full version of the network contains a large fraction of ORFans (genes with no detectable homologs) and rare genes with a patchy distribution, which reduces the signal-to-noise ratio and makes a detailed analysis computationally unfeasible. To overcome these problems, we built a reduced version of the network that only contains core genes, making it computationally tractable and more informative in terms of evolutionary relationships among viruses. In short, core genes were identified based on their low evolutionary loss rates, according to the procedure described in the next section. Table 1 summarizes the basic statistics of the full and core gene networks. With the exception of this section, all results presented here are based on the core gene version of the bipartite network. A visual inspection of the network (Fig. 1A) indicates that (i) viruses from different taxa generally occupy different regions of the network, and (ii) despite the abundance of taxon-specific genes, there is a complex pattern of gene sharing involving, to a greater or lesser extent, all included taxa. It is also notable that *Polyomaviridae* and *Papillomaviridae* (two families of dsDNA viruses with the smallest genomes) and viruses infecting archaea (primarily hyperthermophilic *Crenarchaeota*) form well-defined clusters that are only weakly connected to the rest of the network.

**TABLE 1:** Basic properties of the bipartite dsDNA viral network

Element	Value for full network	Value for core genes^a^
Genomes	1,073	1,071
Gene families	33,793	1,576
Edges	98,343	30,661
Edge density	0.003	0.018
Modules	NA^b^	19
Mean no. of genes per genome	92.1	28.8
Mean gene abundance (mean with ORFan excluded)	2.9 (6.7)	19.3

a The core gene version of the full bipartite network is a bipartite subnetwork that includes core gene families and genomes with at least one core gene.

b NA, not applicable.

In the context of networks, the degree of a node (*k*) is the number of edges connected to that node. In the bipartite network of viruses, the degree of a gene family represents the number of genomes in which the gene family is found, and it follows a power law distribution, *P*(*k*) ∼ *k^−γ^*, with the exponent γ ≈ 2 (Fig. 1B, left). Power law distributions are characterized by having a long tail, which means that the frequency of nodes with high degrees is not negligible. Thus, the power law distribution of the gene degrees implies the existence of hubs, i.e., gene families present in a large fraction of the genomes. Specifically, there are 12 gene families present in at least 20% of the analyzed genomes. In contrast, the number of gene families per genome (i.e., the genome degree) follows a roughly uniform distribution with a bulk that encompasses viruses and MGE encoding from 3 to 100 genes and a tail of highly connected nodes that corresponds to giant viruses (Fig. 1B, right). The flat shape of the degree distribution for genomes indicates that the genome network, in sharp contrast to the gene network, contains few if any prominent hubs: even the largest giant viruses only harbor a small fraction (5% or less) of all gene families.

Another useful topological measure is the clustering coefficient (*C*), which quantifies the extent to which the neighbors of a given node are also connected with each other. For gene families, the bipartite version of the clustering coefficient (38) decays with *k* as *C*(*k*) ∼ *k*^−α^, where α = 1.2 (Fig. 1C, blue). Such a scaling of the clustering coefficient is often considered an indicator of hierarchical modular organization, in which low-level modules of tightly connected nodes join through higher-level nodes to produce larger, less-cohesive supermodules, in an iterative manner (39). In the case of the virus network, low-level modules are cemented by signature genes, whereas hallmark genes, at the top of the hierarchy, bring modules together. This hierarchical structure is not observed for genomes (Fig. 1C, red). Accordingly, the bipartite network appears to be held together primarily by hallmark genes rather than chimeric genomes (see also below).

### Core genes in viral genomes.

**FIG 2:**
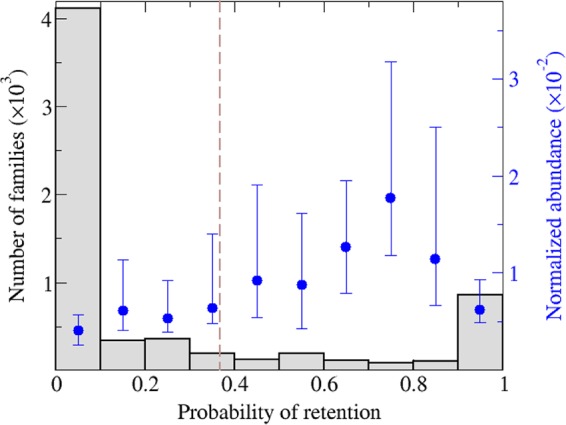
Core-shell-cloud structure of viral gene families. For each bin, the bar indicates the number of gene families with a retention probability in the range defined by the *x* axis. The blue dots indicate the median abundance of such families in the whole set of genomes (error bars correspond to the 25th and 75th percentiles). Family abundances were normalized so that an abundance equal to 1 means that the given family is present in each genome (the contributions of highly similar genomes were downweighted to compensate for sampling bias [see Materials and Methods]). The gene families with the highest retention probability (right-most bin) are typically restricted to a small number of genomes (median abundance, approximately 0.06). In contrast, many of the “core” genes according to the intuitive definition (i.e., those present in a large number of genomes) belong to the bin with a retention probability in the range of 0.7 to 0.8. For the purpose of this work, gene families to the right of the dashed, vertical line (i.e., those with a retention probability greater than 1/*e*) were considered core genes.

Viral gene families show the tripartite core-shell-cloud structure that has been identified as a general feature of gene frequency distributions across the diversity of life forms (40–43). Although there are no universal viral genes, the U-shaped distribution becomes evident when “coreness” is defined as the retention probability of a gene (Fig. 2; see Materials and Methods for details). Figure 2 also shows (in blue) how widespread the genes within each bin are. From this perspective, genes on the left of the distribution are either ORFans or rare genes with patchy distribution patterns. On the right side, genes with retention propensities close to unity are typically taxon-specific genes restricted to a small number of closely related genomes. The most widespread genes, i.e., the core genes according to the intuitive definition, exhibit retention propensities around 0.7 to 0.8. That this value is smaller than unity could be caused by three factors (or any combination thereof): (i) failure to detect all homologs of an ancestral gene in a set of distantly related genomes due to high sequence divergence, (ii) loss of ancestral genes in some lineages, or (iii) occasional transfer of a lineage-specific core gene to unrelated lineages where it does not tend to be retained, thus affecting the net estimate of its retention probability (although this effect is minimized by setting a similarity threshold to calculate the loss rates; see also Materials and Methods).

We defined core genes as those with a loss rate below unity [i.e., retention probability greater than exp(−1)]. Application of this criterion yielded 1,576 core gene families (see Table S1 in the supplemental material), which represented about 5% of all the families in the data set. Table 2 lists the top 25 core genes, which include some of the most conspicuous viral hallmark genes that have been previously identified qualitatively. The abundances of these genes were computed by adding the similarity-weighted contributions of genomes that contain a given gene and normalized with respect to the total number of genomes. Remarkably, this list encompasses two major classes for each of several viral hallmark proteins that are responsible for the key functions in virion morphogenesis and genome replication, in particular, icosahedral capsid proteins, DNA-packaging ATPases, DNA polymerases, and primases-helicases. The ranking of the core genes reflects the diversity of the groups of viruses in which these genes are most common. Due to the fact that tailed bacteriophages (order *Caudovirales*) are the most diverse (and most abundant) group of viruses on earth (2, 44, 45), the top four core genes are primarily represented in these phages. The core set combines genes that are limited in their spread but appear to be essential and are never lost in the respective groups of viruses (although in some cases could be missed due to the extreme sequence divergence), such as packaging ATPases or phage portal proteins, and genes that are represented in highly diverse groups of viruses but are lost relatively often, such as helicases (Table 2). Extending the latter trend, there are some noncore genes that are highly abundant and spread over diverse groups of viruses but failed to make it to the core set due to their high loss rates; the most widespread of these “viral mobilome” genes are listed in Table 3.

**TABLE 2:** Top 25 core genes sorted by normalized abundance

Family no.	Annotation^a^	Retention^b^	Abundance^c^	Taxon(s) with presence
5	**Terminase, large subunit**	0.969	0.661	*Herpesvirales*, *Caudovirales*
13	**Major capsid protein, HK97-like**	0.960	0.532	*Herpesvirales*, *Caudovirales*
10	**XRE-family HTH domain**	0.718	0.327	*Caudovirales*
16	**Portal protein**	0.740	0.314	*Caudovirales*
8	DEAD-like helicase	0.522	0.298	Megavirales, *Caudovirales*
6	**DNA primase/helicase (DnaB)**	0.683	0.290	*Caudovirales*
11	**DNA polymerase B**	0.886	0.255	Adenoviridae, “Megavirales,” Polintons, some virophages, *Baculoviridae* and related viruses, *Herpesvirales*, *Tectiviridae*, mitochondrial and cytoplasmic plasmids, *Ampullaviridae*, Salterprovirus, some *Caudovirales*
24	**Integrase**	0.794	0.254	*Caudovirales*
111	**Protease (herpesvirus S21, phage U9/U35)**	0.379	0.254	*Herpesvirales*, *Caudovirales*
22	**Bacteriophage HK97-gp10, putative tail component**	0.904	0.240	*Caudovirales*
4	**D5-like primase-helicase**	0.594	0.240	“Megavirales,” *Baculoviridae*, some *Caudovirales*
27	HNHc endonuclease	0.600	0.222	*Caudovirales*
20	DNA polymerase A	0.763	0.205	*Caudovirales*
19	Ribonucleotide reductase large subunit	0.571	0.204	“Megavirales,” *Herpesvirales*, some *Caudovirales* (mostly *Myoviridae*)
18	Thioredoxin	0.371	0.174	“Megavirales,” some *Caudovirales*
2	Ribonucleotide reductase small subunit	0.440	0.169	“Megavirales,” *Herpesvirales*, some *Caudovirales* (mostly *Myoviridae*)
23	Phage tail tape measure protein	0.690	0.164	*Siphoviridae*, some *Myoviridae*
30	UvrD-like helicase	0.886	0.162	“Megavirales,” *Herpesvirales*, some *Baculoviridae*, some *Caudovirales*
35	Portal protein	0.794	0.158	*Siphoviridae*
12	**A32-like packaging ATPase (FtsK/HerA)**	0.868	0.156	“Megavirales,” Polintons, *Lavidaviridae* (virophages), *Tectiviridae*, *Corticoviridae*, *Turriviridae*, *Sphaerolipoviridae*
26	Phage mu protein F, putative minor head protein	0.612	0.143	*Myoviridae*, *Siphoviridae*
68	**Double jelly roll MCP**	0.786	0.136	*Adenoviridae*, “Megavirales,” Polintons, *Lavidaviridae* (virophages), *Tectiviridae*, *Corticoviridae*, *Turriviridae*
44	Baseplate J family protein	0.895	0.133	*Myoviridae*
36	AAA family ATPase	0.843	0.120	*Bicaudaviridae*, “Megavirales” (no *Poxviridae*), some *Myoviridae*/*Siphoviridae*
47	RuvC Holliday junction resolvase; poxvirus A22 family	0.600	0.117	“Megavirales,” some *Caudovirales*

a Bold text is used to denote hallmark genes.

b The retention probability of a gene family is equal to exp(−*r*), where *r* is the estimated loss rate (see Materials and Methods).

c The abundances were normalized to the total number of genomes, such that a family present in every genome would have an abundance equal to 1.

**TABLE 3:** Representative genes from the viral mobilome (low retention propensity, high abundance)

Family no.	Annotation	Retention^a^	Abundance^b^	Taxon(s) with presence
31	HNH endonuclease	0.002	0.134	*Mimiviridae/Marseilleviridae*, *Phycodnaviridae*, *Caudovirales*
32	dUTPase	0.092	0.158	*Adenoviridae*, “Megavirales,” *Herpesvirales*, *Caudovirales*, *Baculoviridae*
43	HNH endonuclease	0.012	0.103	*Mimiviridae/Marseilleviridae*, *Caudovirales*
48	BRO protein, phage antirepressor	0.118	0.121	*Baculoviridae*, *Poxviridae*, *Ascoviridae/Iridoviridae*, *Caudovirales*
56	DUF3310	0.110	0.124	*Caudovirales*
79	DNA methylase N-4/N-6	0.005	0.109	*Caudovirales*, *Phycodnaviridae*, *Pandoravirus*, *Bicaudaviridae*
80	Peptidoglycan recognition protein	0.049	0.105	*Caudovirales*
91	ssDNA-binding protein, SSB_OBF domain, COG0629	0.111	0.122	*Caudovirales*
30573	Thymidine kinase	0.129	0.178	“Megavirales,” *Caudovirales*

a The retention probability of a gene family is equal to exp(−r), where *r* is the estimated loss rate (see Materials and Methods).

b The abundances were normalized to the total number of genomes, such that a family present in every genome would have abundance equal to 1.

A common debate among researchers in virus evolution is which genes represent the viral “self” and thus are most important for evolutionary reconstruction: those for structural proteins or those for components of the replication machinery (19, 46–52). The present quantification of gene “coreness” seems to resolve this dilemma by showing that members of these two functional categories are mixed in the ranked list of core genes (Table 2; see also Table S1 in the supplemental material), highlighting the equal importance of both categories of genes. Below, we return to some of the core genes when discussing connections between different groups of viruses.

### Modular structure of the viral network.

To unveil the internal structure of the viral network, we applied a module identification algorithm to delineate sets of genomes and gene families that are densely connected to each other. The algorithm is aimed at finding the partition of the network that maximizes Barber’s bipartite modularity (a standard measure of the quality of the modules). Because modularity optimization is a combinatorial problem, it is generally impossible to solve it exactly for large networks. Instead, heuristic or stochastic approaches, such as simulated annealing, have to be used (see Materials and Methods for details), and different runs of the same algorithm can yield variable results due to multiple local maxima. To overcome this difficulty, we ran 100 replicates (realizations) of the algorithm which rendered 100 (not necessarily different) solutions for the module identification problem. Of these, the solution with the highest quality (evaluated as the value of Barber’s bipartite modularity) was taken as the optimal partition, and the rest of the replicates were used to assess the robustness of the modules in that optimal partition. Specifically, for each possible pair of genes or genomes assigned to the same module, we calculated the fraction of replicates in which both elements were grouped together. The average fraction over all pairs was taken as the robustness of the module. Similarly, the cross-similarity between two distinct modules is the fraction of replicates in which pairs of genomes, with one from each of the modules, appear in the same group.

**FIG 3:**
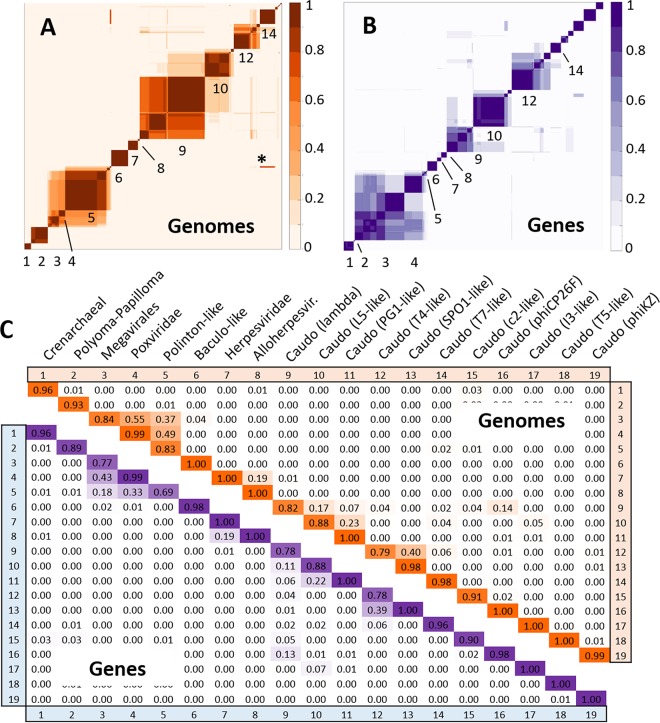
Robustness and cross-similarity of modules in the virus bipartite network. (A and B) Heat map representations of the module robustness matrices for genomes (A) and gene families (B). To generate these matrices, nodes of one class (genomes or gene families) were sorted according to the module they belong to in the optimal partition of the network. For each pair of nodes, the matrix contains the fraction of 100 replicates in which both nodes were placed in the same module. Robust modules appear as blocks in the module robustness matrix; deviations from the block structure correspond to modules that are sometimes merged or nodes without a clear module assignation. The asterisk shows the case of mitochondrial plasmids which belong to module 5 in the best partition but are often assigned to module 14. (C) Quantitative summary of the average robustness of modules at the genome and gene level (elements on the diagonal) and the cross-similarity between pairs of modules (fraction of replicates in which nodes of both modules appear together; off-diagonal elements). See Table 4 for the list of the taxa assigned to each module.

The network showed a significant modular structure, with an optimal partitioning that consisted of 19 modules (*P* < 0.01, compared to the Barber’s modularity of a random network with the same degree distribution) (Table 4; see also Table S3 in the supplemental material for the module composition). The modules generally were highly robust, with respect to both genes and genomes, as determined by analysis of the module composition in alternative network partitions (Table 4; Fig. 3). The number of genomes in the modules spanned 2 orders of magnitude, from 282 in the largest module (module 9) to 3 in the smallest module (module 18). Given that highly similar genomes were clustered prior to network construction, the number of genomes in a module seems to be a meaningful reflection of its heterogeneity. Each of the modules contains multiple core genes, with over 300 genes in the largest (in terms of the number of genes), module 3. However, most of these genes are limited to tight groups of viruses within a module, whereas only a few hallmark genes hold the modules together.

**TABLE 4:** Modules of the dsDNA virus network

Module	Composition (genomes)	Representative gene product(s)^a^	No. of genomes	No. of genes	Robustness^c^	
					Genomes	Genes
1	Crenarcheal viruses except *Turriviridae*	RHH domain-containing proteins **(S)**	27	59	0.98	0.98
2	*Papillomaviridae* and *Polyomaviridae*	Papillomavirus L2 protein **(S)**	70	7	0.93	0.89
3	“Megavirales” (except *Poxviridae*)	D5-like primase-helicase **(H)**	46	304	0.81	0.76
4	*Poxviridae*	Virion core protein P4a, IMV membrane protein, metalloproteinase, poly(A) polymerase **(S)**	26	107	0.99	0.99
5	*Adenoviridae*, *Lavidaviridae* (virophages), Polintons, PLV, *Tectiviridae*, *Corticoviridae*, *Sphaerolipoviridae*, *Turriviridae*, *Salterprovirus*, some mitochondrial plasmids^b^	pDNAP, packaging ATPase (FtsK superfamily), double and single jelly roll capsid proteins, Ulp1-like cysteine protease **(H)**	183	29	0.88	0.77
6	*Baculoviridae*, *Hytrosaviridae*, *Nudiviridae*, *Nimaviridae*, and some cytoplasmic plasmids^b^	*per os* infectivity factors 0–5, capsid protein (p95/vp91) **(S)**, S_TKc serine/threonine protein kinase **(C)**	70	61	1.00	0.98
7	*Herpesviridae*	Envelope glycoproteins H, M, B, UL73; tegument proteins UL7, UL16 **(S)**	41	24	1.00	1.00
8	*Alloherpesviridae*	Capsid triplex protein **(S)**	7	34	1.00	1.00
9	Multiple *Caudovirales*, *Peduovirinae* (P2-like), lambda- like, T1-like, phiC31, mu-like, P22-like, *Picovirinae*, *Plasmaviridae*^e^	HK97-like capsid protein, large terminase subunit, protease, tyrosine integrase **(H)**	282	165	0.80	0.76
10	*Siphoviridae*, mycobacteriophages (L5-like)	Minor tail protein **(S)**, cutinase (lysin B) **(C)**, PGRP (lysin A) **(C)**	107	211	0.88	0.88
11	*Siphoviridae*, mycobacteriophages (PG1-like)	Replicative helicase/primase- polymerase **(S)**	12	23	1.00	1.00
12	Mostly *Myoviridae*, *Tevenvirinae* (T4-like) and unclassified *Myoviridae*; 6 *Podoviridae* (N4-like), *Malacoherpesviridae*^d^	Tail completion and sheath stabilizer protein, baseplate J protein **(C)**	95	196	0.79	0.81
13	*Myoviridae*, *Spounavirinae* (SPO1-like)	Zn-ribbon-containing structural protein, tail tube subunit, tail assembly chaperone **(S)**, tail sheath protein precursor **(C)**	13	40	0.98	1.00
14	*Podoviridae*, *Autographivirinae* (T7-like) and some other minor groups (Bpp-1-like, VP2-like, N4-like)	Head-to-tail connecting protein **(S)**, DNA primase/helicase (DnaB) **(S)**, DNA polymerase A **(C)**	63	63	0.98	0.96
15	*Siphoviridae*, *Lactococcus* phages c2-like and 936 *sensu lato*	Single-stranded DNA-binding protein **(S)**	11	43	0.94	0.94
16	*Siphoviridae*, *Clostridium* phage phiCP26F and related strains	Cytolysin, ferritin-like superfamily protein **(S)**, phage anti- repressor **(S)**, XRE-like regulator **(C)**	4	32	1.00	0.98
17	*Myoviridae*, I3-like mycobacteriophages	Structural proteins, lysin A **(S)**, many uncharacterized proteins **(C)**	4	62	1.00	1.00
18	*Siphoviridae*, T5-like	Tail proteins (Pb3, Pb4, etc.), NAD- dependent DNA ligase, nicking endonuclease **(S)**	3	74	1.00	1.00
19	*Myoviridae*, phiKZ-like	RNA polymerase beta subunit, RNase H **(S)**	5	43	0.97	1.00

a Representative genes are presented based on their classification (in parentheses and boldface) as signature **(S)**, hallmark **(H)**, or connector **(C)** genes.

b Mitochondrial plasmids from *Babjeviella inositovora* and *Debaryomyces hansenii* were assigned to module 5, and those from *Kluyveromyces lactis*, *Lachancea kluyveri*, and *Millerozyma acaciae* belong to module 6, although these assignations were based on a small number of widespread core genes.

c The robustness is equal to the fraction of replicas in which pairs of members of a module were assigned to the same module, averaged over all possible pairs. Two measures of robustness apply to each module, depending on whether pairs of genes or pairs of genomes were considered.

d Malacoherpesviridae lack the tail components listed as representative genes for module 12 (see text for further details).

e The assignment of the only genome from family *Plasmaviridae* to module 9 is based solely on a shared integrase.

Of the 19 modules, 11 include tailed bacteriophages, emphasizing once again that the order *Caudovirales* is by far the largest, most diverse, extremely heterogeneous group of viruses. Most of the phage modules consist of subsets from a particular family (*Siphoviridae*: modules 10, 11, 15, and 16; *Myoviridae*: modules 13, 17, and 19; *Podoviridae*: module 14). However, two modules (9 and 12) include representatives of two or three families of tailed phages. It is well known that tailed phages extensively exchange genes, so the existence of robust modularity is remarkable in itself, indicating that despite this genetic fluidity, there are several partly isolated gene pools in the phage world. Unexpectedly, module 12 also includes, in addition to phages of the family *Myoviridae*, the only known member of the eukaryotic virus family *Malacoherpesviridae*.

Apart from the phages, the viruses of hyperthermophilic *Crenarchaeota* form a single, highly robust module 1, with the notable exception of the family *Turriviridae* (see discussion below). Although crenarcheal viruses contain many genes with no detectable homologs, the existence of a network of shared genes among these viruses has been noticed previously (53). The network analysis described here not only validates this conclusion but also shows that the crenarcheal virus network is a distinct module isolated from the rest of the virosphere. Other than viruses of hyperthermophilic *Crenarchaeota*, archaeal viruses remain poorly sampled, so that the present analysis included only the family *Sphaerolipoviridae*, which belongs in module 5 (see details below), and four euryarchaeal viruses, all of which share the signature genes of *Caudovirales* and were assigned to module 9.

The remaining 7 modules consist, with a single exception, of viruses infecting eukaryotes. As expected, papillomaviruses and polyomaviruses, the smallest known dsDNA viruses that appear to have evolved from ssDNA viruses, replicating via the rolling circle mechanism (31), form a distinct module that is only weakly connected to the rest of the network. The proposed order “Megavirales” (also known as NCLDV), which combines several families of large and giant dsDNA viruses of eukaryotes that primarily replicate in the cytoplasm of the host cell (54–56), is split between two modules that include, respectively, the poxviruses and the rest of the NCLDV. This separation is not particularly surprising because poxviruses are known as a highly derived group of the NCLDV (54). As discussed below, these two modules share several links and join at a higher level of the network hierarchy (see below).

Similarly, the order *Herpesvirales* is split into two modules that include, respectively, the families *Herpesviridae* and *Alloherpesviridae*, and again, are connected at a higher level. Unexpectedly, the third family of the *Herpesvirales*, namely, *Malacoherpesviridae*, has been assigned to a module containing T4-like tailed phages (see below). A separate module consists of several families of viruses that infect arthropods, including *Baculoviridae*, *Nudiviridae*, *Hytrosaviridae*, and *Nimaviridae*. An evolutionary relationship between these virus families has been suspected from previous comparative genomic studies, and it has been proposed that they could be distantly related to the NCLDV (57–59). The network analysis suggests a more complicated picture, as discussed below.

The most notable module, module 5, consists of several groups of viruses and related MGE from all three domains of life, most of which replicate their genomes via the protein-primed replication mechanism and encode the respective variety of the family B DNA polymerases (pDNAP). Previously, it was hypothesized that Polintons, the virus-like large transposons that belong to this module and have been shown to encode two capsid proteins and, accordingly, predicted to form virions (60), evolved directly from bacterial tectiviruses and then gave rise to several diverse groups of eukaryotic viruses and MGE (21, 61). Given the wide spread of the Polintons among eukaryotes and their apparent central role in the evolution of the module and beyond (see below), we denote this module Polinton-like (PL). The delineation of the PL module in the present network analysis is compatible with the previously proposed evolutionary scenario and also adds to the mix, in addition to the bacteriophage family *Tectiviridae*, another phage family, *Corticoviridae*, and archaeal viruses of the families *Turriviridae* and *Sphaerolipoviridae*. The PL module is held together by the pDNAP and a distinct suite of genes involved in virion morphogenesis that encode the major double jelly roll (DJR) capsid protein, the packaging ATPase and, in many viruses, also a minor, single jelly roll capsid protein and a maturation protease (21, 61). The pDNAP is encoded by most viruses in the PL module, with the exception of the archaeal viruses, corticovirus PM2, some virophages, and Polinton-like viruses (PLV). The cytoplasmic and mitochondrial plasmids also encode pDNAP, which is the only link between these small, capsidless genetic elements and the rest of the PL module. Similarly, *Haloarchaeal* viruses of the genus *Salterprovirus* are included in module 5 based solely on the presence of the pDNAP. The DJR major capsid protein (MCP) and the packaging ATPase are present in all viruses of the PL module except for salterproviruses. Thus, the expansive PL module is unified by both morphogenetic and replicative genes that are present in different members of the module, either separately or together. The finding that all these extremely diverse elements form a single module that is robustly supported at the genome level (although less so on the gene level), whereas at the same level of clustering both “Megavirales” and *Herpesvirales* are split, suggests a close relationship and evolutionary coherence among the PL elements.

### Supermodular structure of the dsDNA virus network.

The existence of substantial cross-similarities between some of the modules (Fig. 3C) implies that there is a higher-order structure in the virus network, such that some of the modules can be joined to generate a hierarchy of supermodules. We implemented an iterative method to identify such supermodules by building higher-order bipartite networks in which the two classes of nodes are (primary) modules and genes that are shared by these modules.

**FIG 4:**
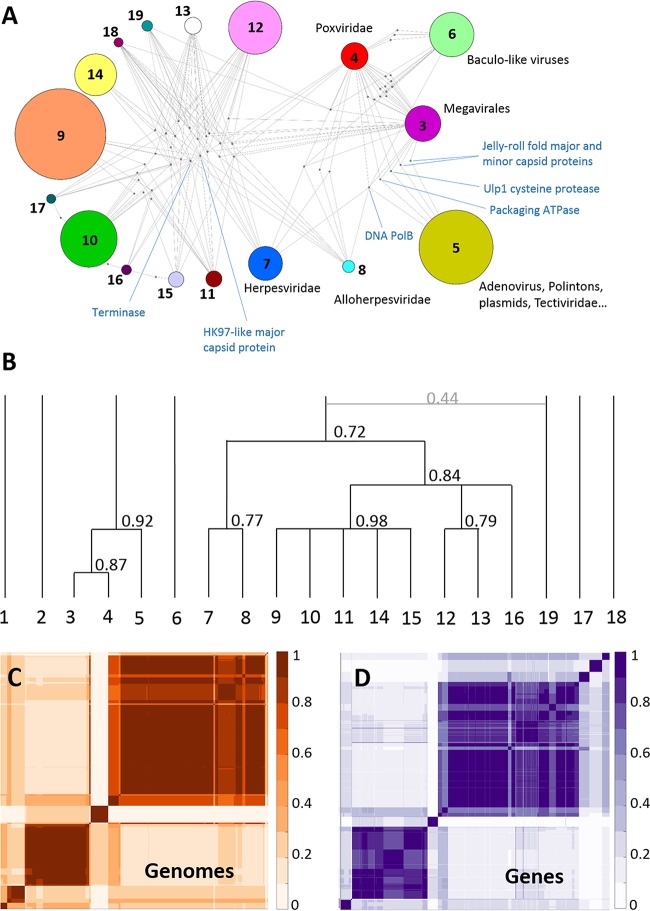
Higher-order structure of the virus network. (A) Bipartite network defined by modules (numbered as for Table 4) and connector genes. A module is linked to a connector gene if the prevalence (relative abundance) of the gene in that module is greater than exp(−1). Modules 1 (crenarcheal viruses) and 2 (polyomaviruses and papillomaviruses) that are only weakly connected to other modules are not represented. Modules are represented as colored circles, with the node size proportional to the number of genomes in the module. Connector genes are represented as dots. The position of some hallmark genes discussed in the text is shown. (B) Tree representation of the hierarchical supermodule structure of the network. At each iteration, two (super)modules were merged if their members clustered together in at least 50 of 100 replicates of the module detection algorithm. Branch lengths are proportional to the number of iterations required for two modules to merge. The number associated to each branch indicates the robustness of the respective supermodule. (C and D) Heat map representations of the supermodule robustness matrices for genomes (C) and gene families (D) after the last iteration of the higher-order supermodule search. To generate these matrices, nodes of one class (genomes or gene families) were sorted according to the supermodule they belong to in the optimal partition of the network. For each pair of nodes, the matrix contains the fraction of 100 replicates in which both nodes were placed in the same supermodule. Robust supermodules appear as blocks in the module robustness matrix.

The first-order module network is depicted in Fig. 4A. Modules representing *Caudovirales* show a dense web of connections, with numerous shared genes, among which the HK97-like major capsid protein and the large subunit of the terminase (the genome-packaging ATPase-nuclease) are the two most prominent ones. These two genes are also shared by the *Herpesvirales*, which additionally connect to some of the *Caudovirales* modules through the S21-U9/U35 capsid maturation protease, the two subunits of the ribonucleotide reductase, and some other genes. The “Megavirales” also share genes with modules containing *Caudovirales*, such as the ribonucleotide reductase and some helicases. The PL module is connected to the “Megavirales” via five connector genes, namely, DNAP (at this level, all family B DNAPs, both protein primed and RNA primed, merge into a single gene cluster), A32-like packaging ATPase, Ulp1-like maturation protease, double jelly roll major capsid protein, and single jelly roll minor capsid protein. Notably, in the bipartite network, all these genes are assigned to the PL module, which appears to be compatible with the proposed central role of Polintons in the evolution of dsDNA viruses of eukaryotes (21, 31). The complete list of connector genes is provided in Table S2 in the supplemental material.

At the highest order of the network hierarchy, the dsDNA viruses form 5 major supermodules: (i) crenarcheal viruses (except *Turriviridae*), (ii) *Polyomaviridae-Papillomaviridae*, (iii) PL elements-“Megavirales,” (iv) *Baculoviridae* and the related families of arthropod viruses, and (v) *Caudovirales-Herpesvirales* (Fig. 4B to D). There are also three minor modules encompassing I3-like, T5-like, and phiKZ-like phages that remained isolated (although the latter join the main *Caudovirales* supermodule in 44 of the 100 replicates). The most notable result is the unification of the PL elements and the “Megavirales” (including poxviruses) into a single module with high robustness (0.92), in agreement with the evolutionary scenario that has been proposed primarily on the basis of the shared morphogenetic genes. In parallel, *Herpesvirales* merge with *Caudovirales* in a moderately supported supermodule (robustness of 0.72).

### Dissection of the PL-“Megavirales” supermodule.

We then sought to investigate in greater detail the internal structure of the large and heterogeneous supermodule that joins the “Megavirales” with the PL elements. Given the shared genes and proposed evolutionary relationships between the Baculo-like viruses and the “Megavirales,” their persistent separation in the full network analysis appeared unexpected, so we included the Baculo-like module in this additional analysis in an attempt to better characterize its relationships with the other modules. The technical advantage of analyzing (super)modules separately is that there is a resolution limit for the module detection algorithm in very large networks (34). Therefore, limiting the scope of the analysis to a subnetwork of particular interest can reveal features of its internal structure that escape the analysis of the full network.

**FIG 5:**
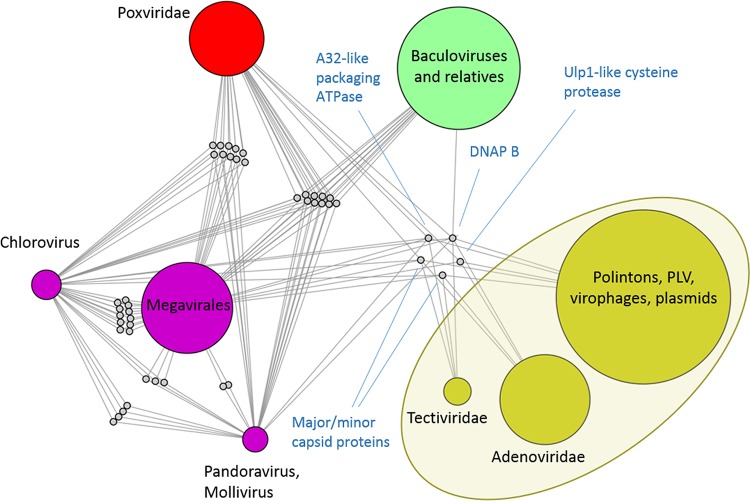
The internal structure of the PL-“Megavirales” supermodule. A module is linked to a connector gene if the prevalence of the gene in that module is greater than exp(−1). Modules are represented as larger circles, with sizes proportional to the number of genomes in the module; colors coding is the same as in Fig. 4. Connector genes are represented as smaller gray nodes. The PL elements, which originally formed a single module (shaded oval), were further dissected to produce the submodule structure shown. The hallmark genes are labeled.

The analysis of the internal structure of the PL-“Megavirales” supermodule produced a new partitioning (Fig. 5; see also Table S3 in the supplemental material), in which the PL module remained unchanged. In contrast, the “Megavirales” module split into *Poxviridae* (which was already identified as a separate module in the initial analysis of the full network), *Pandoravirus-Mollivirus*, a group of five phycodnaviruses of the genus *Chlorovirus* (with Paramecium bursaria chlorella virus as the prototype), and a large module that included the remaining members of the “Megavirales.” The baculoviruses and their relatives remained a separate module as in the full network.

We further analyzed the internal structure of the PL module and identified three submodules: (i) Polintons, PLV (a recently discovered group of Polinton-like viruses [62]), virophages, cytoplasmic plasmids, and one tectivirus (enterobacterial phage PRD1, which lacks several genes that are conserved in the other members of the family *Tectiviridae*), (ii) the rest of the *Tectiviridae* (with *Bacillus* phages AP50 and Bam35 as representative species), (iii) *Adenoviridae* (Fig. 5). The first submodule is the largest and most heterogeneous, as indicated by a relatively low robustness (86/100 compared to 100/100 for the rest of the modules). The submodules are connected through five hallmark genes, the same that maintain the integrity of the PL module as a whole, namely, (i) pDNAP, (ii) A32-like packaging ATPase, (iii) major capsid protein, (iv) minor capsid protein, and (v) Ulp1-like cysteine protease. The first, largest submodule is the only one that harbors all five connector genes, and moreover, they are all assigned to this submodule, again reaffirming the evolutionary centrality of Polintons and the related elements.

The case of baculoviruses and the related viruses of arthropods is of special interest. With the exception of the DNAP, these viruses do not connect directly to the PL module (although there is a small fraction of genomes in the PL group, namely, some virophages, Polintons and PLV, that possess a D5-like primase-helicase, this gene does not constitute a connector with the whole module). Instead, the Baculo-like module shows multiple connections with “Megavirales” and *Poxviridae*. Most of the shared genes are assigned to the Baculo-like module, apparently because they are strictly conserved in this small module but not in “Megavirales.” These shared genes include the two large subunits of the RNA polymerase, mRNA capping enzyme, transcription factor TFIIS and thiol oxidoreductase. Baculoviruses and their relatives also harbor genes assigned to other modules and shared by more diverse groups, such as PolB, ribonucleotide reductase (small and large subunits), S_TKc serine/threonine protein kinase, and D5-like primase-helicase. Despite the clear relationship with “Megavirales,” the lack of jelly roll fold capsid proteins, packaging ATPase of the FtsK/HerA family and Ulp1 cysteine protease, all of which are the connectors within the PL-“Megavirales” supermodule, explains why baculoviruses and their relatives stay as a separate module even at the highest hierarchy level.

### Modularity and interconnections within the order *Caudovirales*.

**FIG 6:**
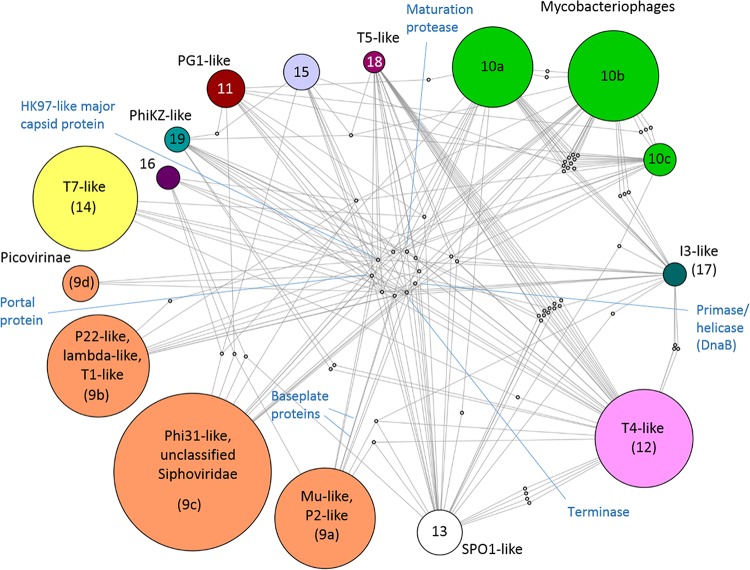
Internal structure of the *Caudovirales* supermodule. A module is linked to a connector gene if the prevalence of the gene in that module is greater than exp(−1). Modules are represented as larger circles, with sizes proportional to the number of genomes in the module; color coding is as shown in Fig. 4. Module 15 contains *Siphoviridae* from the *Lactococcus* phage 936 *sensu lato* and c2-like groups. Module 16 conatins *Clostridium* phage phiCP26F and related strains. Connector genes are represented as smaller gray nodes. Hallmark genes are labeled.

Tailed bacteriophages constitute the most abundant and diverse group of dsDNA viruses (and all viruses). In order to obtain a more precise picture of their module structure and interconnections, we carried out a detailed analysis of the *Caudovirales* subnetwork (Fig. 6; see also Table S3 in the supplemental material).

This reanalysis rendered a finer-grained network structure in which module 9 split into four submodules: 9a, with members of the family *Myoviridae*, such as Mu-like and P2-like phages; 9b, with lambda-like phages and other members of the family *Siphoviridae* (e.g., T1-like and N15-like phages) as well as P22-like phages; 9c, with Phi31-like phages and numerous unclassified siphoviruses; 9d, containing subfamily *Picovirinae* and related unclassified members of the family *Podoviridae*. Two signature gene families are associated with submodule 9a: phage tail protein D and baseplate assembly protein V. In contrast, submodules 9b and 9c are more heterogeneous, and their integrity is maintained by a network of gene sharing which involves multiple tail components and cell lysis proteins. With the exception of the *Picovirinae*, submodules within module 9 are characterized by moderate to high prevalences (relative abundances) of an integrase and a protein with a helix-turn-helix DNA-binding domain of the Xre family. In agreement with the high prevalence of these genes, a survey of the lifestyle of phages in module 9 shows that it is mostly composed of temperate phages (96 of the 110 based on available data; binomial exact test, *P* < 10^−4^).

The large mycobacteriophage module 10 also splits into three submodules, each with a distinct set of signature genes (although the large majority of these genes are poorly characterized). Taking the classification in the Phamerator database (63) (http://phagesdb.org) as a reference, submodule 10a contains L5-like and related phages (Phamerator cluster A), submodule 10b corresponds to clusters E to G and I to P, and submodule 10c includes members from clusters D, H, and R. The phages in these submodules, as well as those in module 11 (PG1-like mycobacteriophages, Phamerator’s cluster B), share a set of minor tail proteins, a peptidoglycan recognition protein (lysin A), and a cutinase (lysin B). The last two genes, required to lyse the mycolic acid-rich outer membrane of the *Mycobacterium* host (64), also appear in the taxonomically unrelated I3-like mycobacteriophages (module 17, Phamerator cluster C). Notably, all phages from submodule 10c, as well as I3-like phages, share the gene coding for the bacterial DNA polymerase III alpha subunit. Although experimental data on the lifestyle of mycobacteriophages are scarce, the high prevalence of an integrase in submodule 10b suggests that most of the phages from this subgroup are temperate.

The genomes of I3-like phages (module 17) contain 6 to 10 genes (approximately 10% of their total gene content) assigned to submodules within the mycobateriophage module 10. Thus, although the two modules are not similar enough to merge, there seems to be a relationship between I3-like phages and other mycobacteriophages that likely reflects gene exchange, especially given that these phages share related hosts.

Of the three modules (9, 12, 14) for which there is enough information about the lifestyle of their members, modules 12 and 14 show a significant predominance of virulent phages (9 of 9 [*P* < 10^−4^] and 13 of 19 [*P* = 0.002], respectively). Of these two modules, the former corresponds to T4-like phages, whereas the latter includes T7-like phages and some other minor groups from the family *Podoviridae*. The reanalysis of the *Caudovirales* subnetwork resulted in refinement of the T4-like module, with approximately 30 unclassified *Myoviridae* moved to module 9a, and resulting in a robustness of 0.99 (compared to 0.79 in the original module reported in Table 4). Signature genes for this refined module include a set of neck, tail, and baseplate proteins, DNAP sliding clamp and clamp loader, and RNAP sigma factor. Module 14, in contrast, is defined by the central position of the head-to-tail connector protein, which is a signature of T7-like viruses and other phages of the family *Podoviridae*. A single-subunit RNAP is also characteristic of this module, although some phages within the module lack it. Finally, two major connector genes, the DNAP A and the DnaB primase-helicase, are also assigned to module 14.

**FIG 7:**
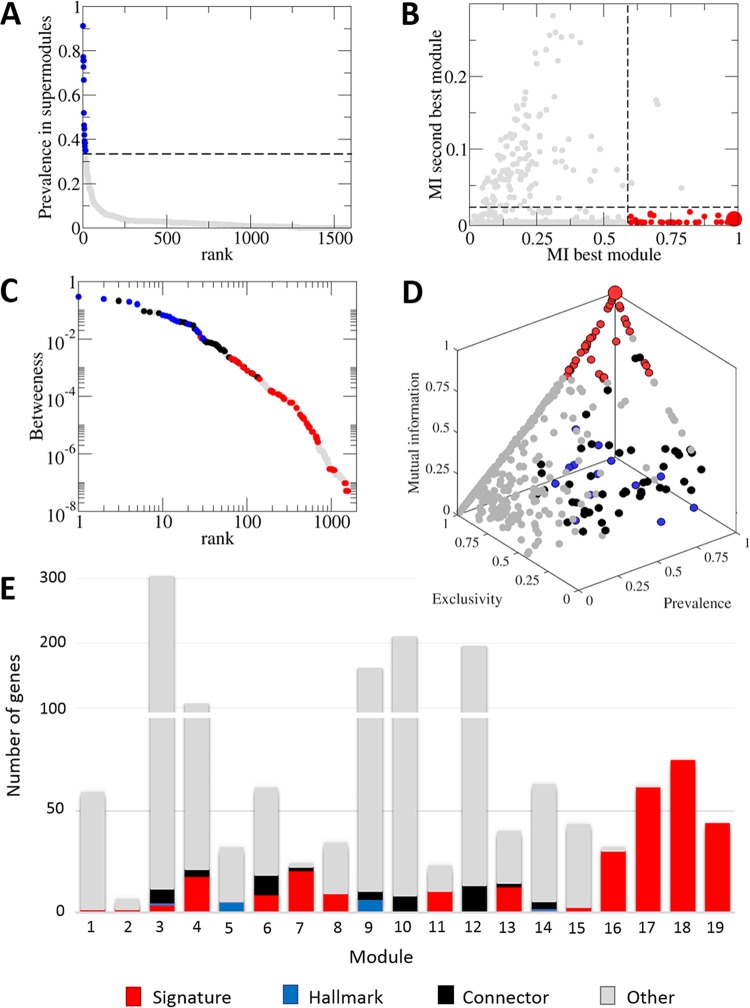
Characterization of viral hallmark genes and module-specific signature genes. (A) All core gene families sorted by their relative prevalence in the major supermodules are shown in gray. Hallmark genes are those that, besides belonging to the set of connector genes, have a relative prevalence greater than 0.35 in at least one of the two major supermodules. (B) Signature genes are those genes with mutual information greater than 0.6 to their best-matching module (*x* axis) and less than 0.02 to their second match (*y* axis). The rest of the gene families are represented in gray for comparison. (C) Betweenness-rank distribution for genes in the bipartite network. The nodes with the highest betweenness correspond to hallmark and other connector genes. Signature genes are represented in red. (D) Three-dimensional representation of core genes based on mutual information, relative prevalence, and exclusivity with respect to their assigned module (same color coding as in panel C). (E) A histogram with the number of signature, hallmark, connector (nonhallmark), and other (gray) genes per module. Reanalysis of the *Caudovirales* subnetwork detected 13 signature genes for module 12, which are not shown in the figure. In panels B and D, a large red point indicates the existence of 205 signature genes whose presence-absence patterns perfectly match their assigned modules.

Of the remaining modules, module 13 consists of SPO1-like phages, module 15 includes *Lactococcus* phages from the c-like and 936 *sensu lato* groups, and the small modules 16 to 19 correspond, respectively, to *Clostridium* phage phiCP26F (and related strains), I3-like mycobacteriophages, T5-like phages, and phiKZ-like phages. These modules, with the exception of the *Lactococcus* phage one, possess large sets of module-specific signature genes (Fig. 7), most of which remain uncharacterized (but see Table 4 for a list of some of these genes).

As shown in Fig. 6, global connections across modules occur through hallmark genes, such as the HK97-like major capsid protein, portal protein, maturation protease, and DNA primase-helicase of the DnaB family. Additionally, baseplate proteins J and W connect modules within the family *Myoviridae*. On a local scale, there are two sets of interconnections: (i) those that bring together mycobacteriophages from submodules 10a, 10b, 10c and, to a lesser extent, I3-like phages, as discussed above; (ii) those that involve T4-like and T5-like phages. Hallmark genes excluded, there are 13 connector genes shared by T5-like and T4-like phages, of which many are related to nucleotide metabolism (multiple nucleotide and nucleoside kinases) and DNA repair (DexA exonuclease, SbcCD repair exonuclease, RNase H).

Given the multiple connections to other modules, it is clear that I3-like, T5-like, and phiKZ-like phages are bona fide members of the *Caudovirales* network. Therefore, it is surprising that they do not join the major bacteriophage supermodule, even at the highest level of hierarchy. The members of these modules have large genomes (phiKZ-like, 211 to 317 kb; T5-like, 111 to 122 kb; I3-like, 153 to 165 kb) which are characterized by a significant number of conserved genes without clear homologs outside the module itself (65). Our analysis showed that more than 75% of the genes that are highly retained in these genomes are module-specific signature genes. In terms of the network, the fact that these modules stay separated from the rest reflects this distinctiveness of their genome content. In contrast, network analysis places the large *Vibrio* phage KVP40 (245 kb) in the T4-like module, as widely accepted, and it further merges the T4-like module (also characterized by large genomes but with only 15% of signature genes) with the major *Caudovirales* supermodule.

### Orphan modules.

A closer inspection of the modules that remain unmerged showed that crenarcheal viruses do not form strong connections with the rest of the dsDNA virosphere, although several genes are shared with individual families within other modules. The RHH DNA-binding domain that is typical of archaeal viruses also appears with low prevalence in module 5 (PL elements), because the archaeal virus families *Turriviridae* and *Sphaerolipoviridae* are assigned to that module. Archaeal viruses of the family *Bicaudaviridae* encode an AAA family ATPase that is common in “Megavirales” and is also present in T4-like phages. Similarly, archaeal viruses of the family *Lipothrixviridae* encode a DEAD-like helicase that is shared with members of the “Megavirales” as well as some tailed phages. Finally, viruses of the family *Ampullaviridae* encode a pDNAP.

Viruses of the families *Polyomaviridae* and *Papillomaviridae* connect with the other modules solely through the SF3 helicase domain that is present in the DNA replication proteins of these viruses (the large T-antigen of polyomaviruses and E1 protein of papillomaviruses). However, this protein as a whole (fusion of the N-terminal origin-binding domain and the C-terminal SF3 helicase domain) has no orthologs among other dsDNA viruses but rather is a derivative of Rep proteins of ssDNA viruses (31). The capsid protein of polyomaviruses and papillomaviruses is of the single jelly roll variety but shares only extremely distant similarity to the minor capsid proteins of PL elements and “Megavirales.”

### Unexpected connections and hybrid viral genomes.

The network analysis revealed the hybrid nature of several groups of viruses that possess combinations of core genes from different modules as well as unexpected splits of some supposedly well-established groups. The baculoviruses and their relatives are perhaps the prime case in point, as discussed above, but there are several other notable examples of virus groups with a “split personality.”

For instance, the archaeal viruses in the family *Turriviridae* encode RHH domain proteins that are typical of other archaeal viruses but merge into the PL module with the eukaryotic viruses due to the shared genes for the packaging ATPase and major and minor jelly roll capsid proteins. In a similar vein, viruses of the archaeal family *Ampullaviridae* encode a pDNAP but are assigned to the crenarcheal module based on their sharing of a glycosyl transferase with other archaeal viruses.

Mitochondrial plasmids, which in the best solution are assigned to the PL module, often join module 14 (T7-like, *Autographivirinae*). These elements possess two core genes, namely, pDNAP and a single-subunit phage-type RNAP that, respectively, link them to the PL module and the T7-like module. Because of this hybrid composition, module assignment of these plasmids is uncertain.

Among the cytoplasmic plasmids, three were assigned to the Baculo-like module, whereas the rest were assigned to the PL module. Those plasmids that joined the baculoviruses and their relatives possess four core genes, namely, pDNAP, DEAD-like helicase (assigned to *Caudovirales* but common also among “Megavirales” and poxviruses), mRNA capping enzyme, and the largest subunit of RNAP (both assigned to baculoviruses but also nearly ubiquitous in “Megavirales” and represented in all poxviruses). This mix of widespread genes causes the split of the plasmid group between two modules, emphasizing the network character of the evolution of selfish elements.

The order *Herpesvirales* is split between two compact modules that duly combine within a supermodule (Fig. 4) and the phage module 12 (mostly *Tevenvirinae*), which includes the only available genome from the family *Malacoherpesviridae* (66). This herpesvirus possesses 6 core genes, namely, DNAP (module 5), large subunit of the terminase (module 9, *Caudovirales*), large and small subunits of the ribonucleotide reductase (assigned to module 12, *Tevenvirinae*, although also abundant in “Megavirales,” poxviruses, *Herpesvirales*, and some other phage modules), RNA ligase (assigned to *Tevenvirinae* but also common in “Megavirales”), and RING finger-containing ubiquitin ligase (assigned to baculoviruses, also present in poxviruses and some “Megavirales”). Although Malacoherpesvirus shares only a few core genes with *Herpesvirales*, its clustering with *Tevenvirinae* is solely based on genes that are widespread among diverse viruses. Thus, this grouping appears to be an artifact caused by the small number of core genes and the current lack of diversity among the malacoherpesviruses. It seems likely that once more genomes from this group become available, additional genes shared with *Herpesvirales* will be identified, likely resulting in a change of the module affinity.

Among the tailed bacteriophages, it is worth dissecting the case of the N4-like phages that are split between the T7-like and T4-like modules. Genome analysis shows that N4-like phages contain a mix of genes characteristic of each module; they share with T7-like phages the head-to-tail connecting protein, DNAP A, and a single-subunit phage-type RNAP. In contrast, UvrD-like helicase as well as rIIA and rIIB proteins are shared with T4-like phages. This chimeric gene composition of N4-like phages makes it difficult to assign them to a single module; indeed, the main difference between those assigned to the T4-like and T7-like modules is the presence or absence of thioredoxin and ribonucleotide reductase, both formally assigned to the T4-like module, although widespread among bacteriophages and eukaryotic viruses.

Members of the *Picovirinae* (phi29-like bacteriophages) also appear to be chimeric entities that encode a genome replication machinery shared with the viruses and related MGEs of the PL module, whereas virion structure places them among the *Caudovirales*. Indeed, phi29-like phages encode bona fide HK97-like major capsid proteins (67), a packaging ATPase that is a distant homolog of the large subunit of the terminase of other tailed phages as well as the portal protein (68). Thus, the phi29-like phages apparently evolved via recombination between a tailed phage and a tectivirus encoding a DJR capsid protein and pDNAP. The tailed phage contributed the genes for the major capsid protein, portal, and some additional proteins involved in virion morphogenesis and host recognition, whereas the tectivirus provided the linear genome scaffold, including the genes for pDNAP and the terminal protein.

Finally, the family *Sphaerolipoviridae* is peculiar in that it includes bacterial as well as euryarchaeal viruses (Alphasphaerolipovirus and Betasphaerolipovirus infect halophilic archaea, whereas Gammasphaerolipovirus infects bacteria) (69). All members of this family encode an A32-like ATPase and two major capsid proteins (typically called the small and large MCP), which correspond to two halves of the double jelly roll capsid protein characteristic of the “Megavirales”-PL supermodule. Thus, in viruses with the DJR capsid proteins, the pseudohexagonal capsomers are formed from homotrimers of one capsid protein, whereas in sphaerolipoviruses similarly shaped capsomers are heterohexamers of the small and large major capsid proteins (70, 71). Although these MCPs were treated as distinct gene families, sphaerolipoviruses joined the PL module through the packaging ATPase. This assignment appears consistent with the previous suggestion that sphaerolipoviruses diverged from the common ancestor shared with other viruses in the PL supermodule prior to the radiation of the major groups of viruses with the DJR capsid proteins (48). It is worth noting, however, that bacterial sphaerolipoviruses possess two hallmark genes from the *Caudovirales* supermodule, namely, the integrase and an XRE family helix-turn-helix (HTH) domain-containing protein. Thus, this subset of the sphaerolipoviruses comprises another group of viruses with hybrid genomes.

### Classification of viral core genes: signature, connector, and hallmark genes, the glue of the virosphere.

The viral hallmark genes have been previously defined qualitatively as those genes that are shared by multiple, diverse groups of viruses and have no close homologs in cellular organisms (17). The present analysis allows us to quantify this concept by cataloguing and classifying the genes that connect the nodes (connector genes) in the module network. Specifically, we identified hallmark genes as the connector genes with a prevalence in any of the two major supermodules greater than 0.35 (Fig. 7A; see also Materials and Methods). This tally of the hallmark genes identified the familiar suspects, such as the double jelly roll, single jelly roll, and HK97 capsid proteins, terminases and packaging ATPases of the FtsK superfamily (A32-like), DNAP, two primase-helicase families (D5-like and phage replicative helicase DnaB), and two proteases (Ulp1-like and S21/U9/U35). The list was completed by phage portal and tail proteins, transcriptional regulators of the HTH XRE family, and phage integrases of the tyrosine recombinase superfamily. As suggested previously (17), the number of hallmark genes is small: only 14 hallmark genes identified under the above criteria account for 57% of the connections in the module network.

From the perspective of network theory, hallmark and connector genes stand out by their high betweenness (72): 44 of the 50 genes with the highest betweenness are connector or hallmark genes (Fig. 7C). In addition, although they constitute less than 1% of all core gene families, hallmark genes account for 53% of the total betweenness centrality of the network. This value increases to 89% if all connector genes are considered, although they represent less than 4% of the core gene set. Taken together, these results validate our prevalence-driven approach to identify connector and hallmark genes and highlight the role of hallmark genes in maintaining the integrity of the virosphere.

Additionally, we used mutual information to extract signature genes, i.e., genes whose presence/absence pattern is diagnostic of a module (Fig. 7B). As intuitively expected, signature genes show both high prevalence and high exclusivity among modules (Fig. 7D). As shown in Fig. 7E, the number of signature genes per module is highly variable: some modules, such as those that include poxviruses, herpesviruses, T5-like phages, and phiKZ-like phages, are well defined by large sets of signature genes; others, such as the PL and the heterogeneous lambda-like module 9, are instead characterized by a mix of shared hallmark and connector genes. In topological terms, signature genes can be viewed as module-specific hubs, as opposed to the hallmark genes which represent high-level network hubs. Accordingly, techniques for hub identification and classification would seem suitable for identification of signature genes. However, the small size of some modules strongly limits the node degree of their signature genes to the point that they are hardly detectable as hubs. It is for this reason that we chose an information theoretical approach (mutual information) instead of hub detection techniques to identify such genes.

## DISCUSSION

Using bipartite network analysis, we show here that the dsDNA domain of the virosphere forms an almost fully connected network of gene sharing and that this network has a robust, hierarchical modular architecture. The dsDNA viruses and related MGE comprise the largest part of the virus world that includes the most abundant biological entities on earth, the phages of the order *Caudovirales*, as well as some of the most common viruses infecting eukaryotes, such as members of the putative order “Megavirales.” Furthermore, this is the part of the virosphere for which network analysis is expected to be most informative, given the large number of genes with highly variable abundances and often complex evolutionary histories.

The existence of a distinct modular architecture of the gene-genome bipartite network is far from trivial. In principle, alternative models of the virosphere can be easily envisaged, including either a set of disjointed components, or conversely, a continuous structure without robust modules. The latter possibility might be deemed especially plausible given the apparent fluidity of the virosphere, with many documented cases of gene exchange between diverse viruses as well as between viruses and hosts, and the genomic mosaicism that is particularly characteristic of bacteriophages (63, 73). The modularity of the gene-sharing networks is fairly obvious for some viruses but appears unexpectedly in other cases. For example, given the confident identification of a set of about 40 ancestral genes that, some losses notwithstanding, are represented in most members of the putative order “Megavirales” (36), it is not surprising that these viruses belong to a compact module, with the exception of the *Poxviridae*, a derived group that duly joins the module in the next iteration. In contrast, the robustness of the PL module, especially the inclusion of the archaeal families *Turrividiae* and *Sphaerolipoviridae*, was not at all obvious *a priori*, given the small number of shared genes.

The unification of the PL and “Megavirales” modules into a single supermodule, while compatible with the previously proposed evolutionary scenario (21) is even more striking considering the drastic differences between the genome sizes and gene repertoires between the viruses and MGE in the two modules. Equally nontrivial is the unification of the *Herpesvirales* with the *Caudovirales*, primarily on the strength of the shared capsid proteins, the terminase and the maturation protease.

A major advantage of the bipartite network approach is that it provides for the relationships among two categories of objects to be analyzed within the same formal framework (32, 33). This analysis showed that the coherence and robustness of the major modules and supermodules of the network hinge on the uniqueness of a small subset of the core gene set (less than 1% of the core genes and less than 20% of all connector genes), the 14 hallmark genes that are responsible for most of the intermodule connections. Previously, the hallmark genes have been identified informally by comparative analysis of viral genomes (17). Here, we defined the hallmark genes formally and quantitatively as the most prominent connectors between modules in the viral network. The fact that the small set of hallmark genes dramatically differs from the rest of the viral genes with respect to their betweenness underlies the robust modularity of the network. The hallmark genes include those coding for capsid proteins and enzymes involved in virion morphogenesis, along with genes for replication enzymes, such as DNAPs and primases-helicases. These observations seem to settle the perceived conflict between the “structural” and “replicative” perspectives on virus evolution (48, 51, 52). Analysis of the node degree distributions of the bipartite network for the gene and genome nodes clearly indicates that it is the hallmark genes, and not chimeric viral genomes, that are primarily responsible for the network cohesiveness and modularity.

We further defined the signature genes, i.e., those core genes that showed the highest prevalence within but not between modules and hold together some of the modules (the modules are also supported by distinct combinations of the hallmark and other connector genes). The hallmark and signature genes correspond, respectively, to the “date” and “party” hubs that have been identified previously in other biological contexts, such as protein-protein interaction networks, and recognized as the basis of the dynamic modularity in these networks (74–77).

The results of the network analysis revealed a surprisingly well-structured dsDNA virosphere by robustly partitioning the dsDNA viruses into two major supermodules, PL-“Megavirales” and *Caudovirales-Herpesvirales*, which jointly cover most of the dsDNA virosphere, and two smaller, isolated modules, the crenarcheal viruses and polyomaviruses-papillomaviruses. The small polyomaviruses and papillomaviruses, in a sense, do not rightfully belong in the dsDNA virus domain of the virosphere, given their clear evolutionary relationships with ssDNA viruses (31). The few remaining small modules can be expected to join one of the two supermodules upon expansion of the membership.

The two supermodules are defined primarily by the two distinct morphogenetic machineries, each including distinct, unrelated building blocks of icosahedral capsids, the DJR and HK97-like capsid proteins, respectively, and the accompanying genome packaging ATPases and maturation proteases, which belong to unrelated (proteases) or extremely distantly related (ATPases) protein families. Among the known viruses, the two structural modules have not been observed to recombine, i.e., a DJR capsid protein never combines with a terminase, whereas capsids made of HK97-like proteins never package DNA with the help of an FtsK superfamily (A32-like) ATPase. This strict coupling between the capsid building blocks and packaging motors is likely to have a distinct mechanistic underpinning that remains to be elucidated.

The split of the supermodules along the line separating the morphogenetic machineries of the respective viruses is compatible with the concept of the capsid structure as the “self” of a virus (46). However, the coherence of the supermodules, in particular the PL-“Megavirales” one, also depends on the replication and transcription machineries, in particular DNAP and RNAP, which bring into the module various capsidless elements, such as cytoplasmic and mitochondrial plasmids and those Polintons that lack capsid proteins (19).

The PL-“Megavirales” supermodule is by far the largest, most diverse group of eukaryotic dsDNA viruses that includes viruses and related MGE from all major eukaryotic taxa (except for land plants, some of which, however, bear imprints of past infections by members of the “Megavirales” [78]) as well as two bacterial and two archaeal virus families. The viruses and MGE in this supermodule differ by more than 3 orders of magnitude in genome size and lead vastly different lifestyles, yet they are robustly linked by a distinct set of hallmark and other connector genes as well as multiple signature genes within individual modules. The network analysis supports the central position of the Polintons and related viruses in this heterogeneous supermodule, which conceivably reflects the evolutionary potential of these elements for combining features of viruses and transposons. Notably, the PL module is the only module in the network that brings together viruses infecting hosts from all three domains of cellular life. Among the archaeal viruses, the present analysis primarily includes viruses of hyperthermophilic *Crenarchaeota*, whereas viruses of mesophilic archaea, in particular *Lokiarchaeota*, the likely ancestors of eukaryotes (79, 80), remain poorly characterized. Thus, it is currently unclear whether the Polintons, which form the link between the viruses of prokaryotes and those of eukaryotes in this supermodule, are derived from bacterial viruses (tectiviruses from the mitochondrial endosymbiont) or related archaeal viruses that remain to be identified. Given that the PL module owes its coherence to the presence of the JRC (the most common viral structural protein, if both single jelly roll and double jelly roll forms are considered), the associated small packaging ATPases, and pDNAP, a simply organized enzyme involved in the replication of relatively small DNA genomes, this viral module could be as old as cellular life itself, if not older. Indeed, from the perspective of the primordial virus world, in which capsids could have played a key role in the dissemination of the primitive, virus-like genetic elements (81, 82), the viruses of the PL module appear to be strong candidates for some of the earliest forms.

Most of the available genomes from the *Herpesvirales* represent the family *Herpesviridae*, with a rather homogeneous composition of the core genes. The rest of the herpesviruses, in the less thoroughly characterized families *Alloherpesviridae* and *Malacoherpesviridae*, show major differences in the gene repertoires and either join the *Herpesvirales* in a second iteration or fail to join at all, remaining in one of the phage modules. Regardless, the unification of *Herpesvirales* with *Caudovirales* is highly robust and is about as strong as that between different herpesvirus families. This result draws support from structural and biochemical studies showing that viruses from both *Caudovirales* and *Herpesvirales* execute strikingly similar programs of virion assembly and maturation and employ homologous proteins in the key steps of these processes (83–85). Notably, the *Herpesvirales* comprise the only offshoot of the *Caudovirales* (tailed viruses infecting bacteria and archaea), the most abundant group of viruses altogether, in the eukaryotic world (31). In a sense, herpesviruses that so far have been isolated only from metazoa, primarily vertebrates, can be considered “animal phages.” It is also of note that the four archaeal members of the order *Caudovirales* included in this analysis confidently fall into module 9 together with several groups of tailed bacteriophages (Table 4), possibly testifying to the early diversification of the *Caudovirales*.

Predictably, the majority of the identified modules consist of phages of the order *Caudovirales* that join into a supercluster together with *Herpesvirales* in the second iteration of network analysis. The existence of these robust modules clearly shows that despite the well-known fluidity of the phage genomes (63), the pool of phage genes is compartmentalized. The phage modules do not split along family lines, although several modules contain large subsets of phages from the same family. The modules are associated with distinctive sets of signature core genes, such as the head-to-tail connecting protein for the module that harbors most of the *Podoviridae*, the nicking endonuclease for T5-like viruses, and a set of minor tail proteins for the module that encompasses a large group of mycobacteriophages of the family *Siphoviridae*. On a larger scale, modules with taxonomic or ecological similarity are linked by connector genes, e.g., the baseplate J protein for phages of the family *Myoviridae* and the specialized lysins for mycobacteriophages. The partial lack of family coherence is not especially surprising given that phage families have been identified without any reference to evolutionary relationships (86).

A network approach in bacteriophage classification has been reported previously, including a comparison between phage clusters and gene modules that were defined independently (not as parts of a bipartite network of the type explored here) (87). Despite the technical differences, both approaches render similar pictures, with a core of highly interconnected temperate phages and several peripheral modules which include T4-like phages, T7-like phages, and mycobacteriophages. The two analyses also agree in some specific details, such as the subdivision of the mycobacteriophages and the isolation of phiKZ-like phages. The differences seem to stem from the increase in the amount of the genomic data during the time that elapsed since the previous analysis, which is particularly consequential for smaller groups of phages. Thus, in the study of Lima-Mendez and colleagues (87), T5-like phages, represented at the time by a single genome, appeared as a hybrid between T4-like phages and SPO1-like phages. In contrast, inclusion of two additional members in the present analysis gave rise to a distinct module which, despite the connections to T4-like phages, maintained its identity even at the highest level of hierarchy.

The only distinct module of dsDNA viruses of eukaryotes (besides the polyomavirus-papillomavirus module) that fails to join either the PL-“Megavirales” or the *Herpesvirales-Caudovirales* supermodule includes baculoviruses and related viruses of arthropods. This module shares connections with each of the supermodules but lacks some of the key hallmark genes, in particular either type of the capsid proteins and packaging ATPases, and apparently for this reason, it cannot be assigned to either supermodule. Nevertheless, the strongest connection of these viruses is with the “Megavirales,” suggesting that the Baculo-like module is a highly derived offshoot of the PL-“Megavirales” supermodule that, in particular, has lost the ancestral morphogenetic machinery. It should be noted that the loss of structural and morphogenetic proteins has been observed even among the “Megavirales” themselves, e.g., in Pandoraviruses (37).

The viruses of archaeal hyperthermophiles include several families, each with a number of unique features, and with the notable exception of the *Turriviridae*, form a distinct module in the bipartite network. In sharp contrast with the PL-“Megavirales” and *Herpesvirales-Caudovirales* supermodules, the archaeal module is held together not by hallmark genes coding for key proteins of viral morphogenesis and replication but rather by accessory, regulatory genes, such as those encoding RHH and HTH domain-containing proteins, which are particularly abundant in these viruses (53). These regulatory genes appear to be frequently transferred between archaeal viruses as well as between viruses and hosts, which seems to account for the coherence of this module. The extreme diversity of the structures of the crenarcheal viruses and the near lack of identifiable proteins involved in replication clearly distinguishes them from viruses of bacteria and eukaryotes. The evolutionary processes and pressures that have led to this distinct character of the crenarcheal viruses remain elusive (88). Formally, the family *Turriviridae* links the crenarcheal part of the virosphere with the PL module, but the implications of this weak connection are at present difficult to assess.

The results of the viral network analysis seem to reflect four interrelated but distinct processes that apparently shaped the large-scale structure of the virosphere: (i) vertical inheritance of gene ensembles that define highly cohesive groups of viruses, e.g., the “Megavirales,” (ii) sampling of hallmark and other connector genes from the partially compartmentalized but continuous in space and time pool of MGE that underlies the connectivity and modularity of the virosphere as a network, (iii) horizontal gene transfer between viruses that yields additional intermodule connections, and (iv) capture of host genes by viruses, including multiple, independent acquisitions of homologous genes.

The modules and supermodules of viruses and related MGE represent groups with coherent evolutionary histories that, given the limited applicability of traditional phylogenetic methods to the virosphere, are likely to help further evolutionary studies as well as virus taxonomy. Further developments could include extension of the bipartite network analysis to all viruses and MGE as well as their cellular hosts.

## MATERIALS AND METHODS

### Sequences.

Protein sequences were collected from the NCBI Genome Database for all available genomes of dsDNA viruses with an assigned family. Specifically, we collected genomes belonging to the orders *Herpesvirales* (families *Herpesviridae*, *Alloherpesviridae*, and *Malacoherpesviridae*), *Caudovirales* (families *Siphoviridae*, *Podoviridae*, and *Myoviridae*), *Ligamenvirales* (*Lipothrixviridae* and *Rudiviridae*), the proposed order “*Megavirales*” (families *Ascoviridae*, *Asfarviridae*, *Iridoviridae*, *Marseilleviridae*, *Mimiviridae*, *Phycodnaviridae*, and *Poxviridae*, as well as *Pandoravirus* and *Pithovirus*), the families *Adenoviridae*, *Tectiviridae*, *Ampullaviridae*, *Bicaudaviridae*, *Corticoviridae*, *Fuselloviridae*, *Globuloviridae*, *Guttaviridae*, *Sphaerolipoviridae*, *Turriviridae*, *Baculoviridae*, *Nudiviridae*, *Hytrosaviridae*, *Nimaviridae*, *Papillomaviridae*, *Polyomaviridae*, and *Plasmaviridae*, the genus *Salterprovirus*, and the available virophages (*Lavidaviridae*). The family *Polydnaviridae* was excluded given the extreme divergence of the genomes of these viruses, which consist primarily of host-derived or inactivated viral genes. This data set was complemented by several additional groups of sequences from recently reported yet unclassified viruses or MGE for which evolutionary relationships with dsDNA viruses have been demonstrated. In particular, sequences from virophages YSLV5, YSLV6, and YSLV7, Mollivirus, and Faustovirus were retrieved from the NCBI nonredundant protein database. Sequences of Polintons and Polinto-like viruses, as well as mitochondrial and cytoplasmic dsDNA plasmids, were added by using previously described custom data sets. In total, the initial data set contained 137,331 sequences from 1,442 genomes.

### Automatic classification of genes into homologous families.

First, all protein sequences were clustered at 90% identity and 70% coverage by using CD-HIT (89) to generate a nonredundant data set. For each sequence in this set, a BLASTp search (90) was carried out against all other included sequences, and hit scores were used to generate a sequence similarity network. This procedure involved two steps: initially, a BLASTp search with composition-based statistics (91) and filtering of low-complexity regions was used to determine valid hits (e-val cutoff of 0.01, database size fixed to 2e7). Scores for those hits were subsequently collected from a BLASTp search with neither composition-based statistics nor a low-complexity filter. Sequences with best (reciprocal) hits to other sequences from the same genome were combined and treated as in-paralogs.

The set of BLAST hits defines a weighted sequence similarity network in which nodes are sequences and edges connect significantly similar sequences, with a weight proportional to the hit score. Preliminary groups of homologous genes were identified as communities in the sequence similarity network. In the context of network theory, a community is a set of nodes that are densely interconnected compared to the average node degree of the network. To find such communities, we employed the Infomap software (92) (100 trials, 2-level hierarchy). The communities of sequences in the sequence similarity network tend to constitute partitions of larger homologous groups, with sequences from distantly related taxa often located in separate communities. Therefore, we applied profile analysis to merge communities that consisted of homologous sequences. Sequences in each community were aligned with Muscle (93) (default parameters); the alignments were used to predict secondary structure and build profiles with the tools “addss” and “hhmake” available within the HH-suite package (94). Profile-profile comparisons were carried out using HHsearch (95). Hits were accepted or rejected based on their probability, relative coverage, and length. Specifically, hits with a probability greater than 0.90 were accepted if they covered at least 50% of the length of the profile; additionally, hits with a coverage of 20% or greater were also accepted if their probability was greater than 0.99 and their length was greater than 100 amino acids. The choice of this heuristic filtering strategy was motivated by its performance on benchmark collections of viral orthologous genes (POGs [9] and NCVOGs [37]). This pipeline rendered a total of 33,980 clusters of homologous sequences, of which 11,950 comprised multiple sequences and 22,030 were singletons (ORFans).

### Manual curation.

Some homologous proteins shared among highly diverse groups of viruses (e.g., capsid proteins) have diverged to the point of becoming inaccessible to the currently available automatable sequence analysis approaches, although their homology can still be demonstrated on a case-by-case basis, through analysis of sequence motifs and structural similarity. Thus, we relied on the previous findings on these highly diverged homologous proteins to manually curate our collection of homologous sequences. The main groups that had to be manually merged included the major capsid proteins with the double jelly roll fold, their associated minor capsid proteins (pentons), Ulp1-like cysteine proteases (21, 60, 61, 96), capsid proteins with the HK97-like fold (50, 85), herpesvirus protease S21 and homologous bacteriophage prohead proteases U9 and U35 (97), and the set of proteins shared among *Baculoviridae*, *Hytrosaviridae*, and *Nimaviridae* (57, 98).

The concept of orthology is readily applicable to groups of viruses with similar overall genome organization, such as the NCLDV or the Polinton-like viruses and MGE. Methods for identification of orthologous genes clusters have been implemented in the construction of databases such as POGs (9, 35) and NCVOGs (36, 37), which were employed as reference sets for the present analysis. The meaning of orthology is less obvious when it comes to comparisons between widely different groups, e.g., NCLDV versus *Caudovirales*. Nevertheless, an effort was made to adhere to the notion of orthology as closely as possible by including in the same group only homologous genes with analogous functions, for example, capsid proteins or packaging ATPases. Accordingly, hits that were due to promiscuous conserved domains, e.g., P-loop ATPases, were considered spurious for the purpose of this analysis and discarded at this stage. In particular, manual curation involved splitting two groups that had been erroneously merged based on profile-profile comparison. The first of those false hits connected A32-like packaging ATPases to thymidine kinases (both proteins contain the P-loop NTPase domain); the second false hit involved mRNA capping enzymes from “*Megavirales*” and DNA ligases from *Caudovirales* (again, both enzymes contain homologous nucleotidyltransferase domains).

Henceforth, we use the term protein family to refer to the manually curated groups of homologous sequences.

### The bipartite network of viruses.

The bipartite network of viruses was built by connecting genome nodes to protein family nodes whenever a genome contained at least one representative of a given protein family. To avoid redundancy, genomes that shared more than 90% of their protein families (including ORFans) were treated as a single pangenome. The resulting bipartite network contained a giant component, including all genomes except for two Polintons which together with 5 protein families constituted their own minor components. For subsequent analyses, only the giant component was considered.

In the context of the bipartite network, a singleton is a protein family that only appears in one genome (or the pan-genome of a set of 90% similar genomes).

### Identification of core genes.

We adopted an intuitive definition of core genes as those genes that tend to be maintained in a genome for long periods of time, i.e., those with the lowest loss rates in a pure-loss evolutionary model. Let us consider a simple model in which, starting from a common ancestor, the similarity at the family content level diverges uniformly in time according to the differential equation $\frac{dS_{ij}}{dt}=-\frac{S_{ij}}{\sqrt{N_{i}N_{j}}}$, where *S_ij_* is the number of families shared by genomes *i* and *j* and *N_i_* and *N_j_* are the numbers of families in each genome. The geometric mean in this expression aims at biasing the denominator toward the smaller genome, thus avoiding artifacts caused by giant viruses (34); as it has been shown for the “Megavirales,” similar results can be obtained with min(*N_i_*, *N_j_*) as the normalization factor (33). This simple evolutionary model generates a natural distance between any pair of genomes: $D_{ij}=-\text{ln}\left(S_{ij}/\sqrt{N_{i}N_{j}}\right)$. Although a rigorous derivation of this expression from the underlying model requires that *N_i_* and *N_j_* remain constant, the same compositional metrics has been also applied to more general cases (e.g., analysis of evolutionary relationships within the “Megavirales”). The use of this metrics is additionally supported by the fact that the distance-based tree for the “Megavirales” that it generates shows a good correlation with the tree obtained from the concatenated sequences of the “Megavirales” universal core genes (34). For a particular protein family, the probability that it is present in any pair of genomes conditioned on its presence in the last common ancestor is $P_{11}=e^{-r\;\;D_{ij}}/Z$, where *r* is the loss rate of the family relative to the average divergence rate of genomes. Similarly, the probability for the family being lost in only one genome of the pair is $P_{10}=2\;e^{-rD_{ij}/2}(1-e^{-rD_{ij}/2})/Z,$ where *Z* = *P*_10_ + *P*_11_ is a normalization factor. Note that, in a pure loss model, the fact that one of the genomes contains a family implies that the last common ancestor also contained it. However, pairs of genomes that lack any representative of a family have to be discarded, because there is no guarantee that the family was present in their common ancestor. We used the expressions for *P*_10_, *P*_11_, and *D_ij_* to calculate a maximum likelihood estimate of the family-specific loss rate *r*. The presence of one or a few shared families in otherwise unrelated genomes due to HGT could bias loss rate estimates, thus we only considered those pairs of genomes with distances *D_ij_* < 1. This condition implies that both genomes in the pair have to share more than 35% of their gene content (relative to the size of the smaller genome), a degree of similarity that is unlikely to be due to HGT only. Only those gene families with three or more appearances were considered. Genes with relative loss rate *r* < 1 were assigned to the “core.” Note that different values of this threshold would lead to slightly different lists of core genes. However, because the hallmark and signature genes that underlie the multiscale modular structure of the virus network tend to be highly retained, the results are robust to the particular choice of the loss rate threshold. We additionally tested the consistency of the core gene list by defining viral groups based on the network modules (see below) and recalculating the loss rates separately for each group. The aggregated list of core genes produced in this way showed a high agreement with the original one, and in particular it contained all hallmark and signature genes. The retention probability of a gene family was calculated as exp(−*r*). This expression provides the probability that a family has not been lost after one time unit, with time measured in the characteristic time scale of genome composition divergence.

Gene family abundances were computed based on genome-weighted contributions as previously described (99). The purpose of weighting the contribution of a genome to the abundance of a gene is to compensate for sampling bias by assigning smaller weights to groups of closely related genomes. Distances between pairs of genomes were calculated using the same compositional metrics as described above. In the case of nonoverlapping genomes, a conservative estimate of the distance between them was calculated as $\ln\;\left(1+\sqrt{N_{i}N_{j}}\right)$, which corresponds to the time that would take for an ancestor with one more gene to lose all but that one common gene. The genome-genome distance matrix was used to build a neighbor-joining tree, and genome weights were extracted from the tree following the previously described algorithm (99). Family abundances were obtained by adding the weights of all genomes where the family is present. Therefore, an abundance equal to 1 means that the family is present in all genomes of the data set. The prevalence, or relative abundance, of a gene family in a group of genomes was calculated as the sum of weights of those genomes in the group that harbored the gene family divided by the sum of weights of all the genomes in the group.

### Detection of modules in the bipartite genome: core gene network.

To detect sets of related genomes and gene families, we applied Barber’s definition of bipartite modularity (100) to the bipartite network consisting of genomes and core genes. Simulated annealing heuristics (101) was used to identify the partition of the network that optimizes the magnitude $Q=\frac{1}{L}\sum_{i\in \text{G}}\sum_{j\in F}\left(a_{ij}-p_{ij}\right)\;\text{δ}(m_{i},\;\;m_{j})$, where *L* is the number of links in the network, *G* and *F* represent the sets of genomes and core gene families, respectively, *a_ij_* denotes the existence of a link between genome *i* and gene family *j*, $p_{ij}=k_{i}k_{j}/L$ is the probability that a link exists by chance (with *k_i_* and *k_j_* denoting the connectivities of nodes *i* and *j*), and δ is the Kronecker delta function, which takes the value 1 if nodes *i* and *j* belong to the same module and the value 0 otherwise. The Modular software (102) was used to carry out the modularity optimization. The significance of the resulting partition was evaluated by running 100 replicates of a null model of a randomly generated bipartite network with the same size and the same gene- and genome-degree distributions as the original network. All the results reported here correspond to partitions with a *P* value of <0.01. Because of the ruggedness of modularity landscapes in large, complex networks, the partition found by the module detection algorithm corresponds to only one of possibly many local maxima of modularity (34). To account for this limitation, we ran 100 replicates (realizations) of the algorithm and kept the partition with the highest modularity as the optimal partition. The robustness of the modules in the optimal partition was evaluated by comparing them with the modules obtained in the other 99 alternative partitions.

To detect supermodules, we analyzed higher-order bipartite networks consisting of modules and connector gene families. A gene family was considered a connector between two modules if its relative abundance (prevalence) in both modules was greater than exp(−1). The choice of this threshold was motivated by its correspondence with the expected abundance of a gene with a loss rate equal to 1 after a characteristic unit of time of the gene content divergence process. Prevalence thresholds from 0.35 to 0.5 were also tested, with no qualitative differences. Gene families whose abundance exceeded the threshold in a single module were also kept in the network, although they were not classified as connectors. Supermodules were identified by applying the module detection algorithm to the module-level bipartite network. This process was iterated in order to delineate a hierarchy of higher-order modules. The iterative search continued until no more mergers occurred or nonsignificant values of the modularity were obtained. As with primary modules, 100 independent replicates were carried out in order to assess the robustness of the supermodules.

The internal structures of some sets of modules (PL, “Megavirales,” and Baculovirus; PL alone; and modules that included *Caudovirales*) were further analyzed by extracting from the bipartite network a subnetwork consisting of the genomes assigned to each particular set of modules and the core genes connected (but not necessarily assigned) to them. Modules and connector genes in each subnetwork were identified following the same procedure as in the entire network.

### Hallmark and signature genes.

The intuitive idea of “hallmark genes” was formalized as follows: (i) a hallmark gene must be a connector between first-order modules and (ii) it must be sufficiently prevalent in at least one of the two major supermodules. In order to select a suitable prevalence threshold, we searched for gaps in the distribution of relative prevalences and found a noticeable discontinuity near 0.35, which approximately agrees with the criterion used to define connector genes. Therefore, we adopted 0.35 as the minimum relative prevalence that a connector gene must attain in (at least) one of the major supermodules to be considered hallmark.

Signature genes were defined on the basis of their normalized mutual information with respect to their best and second best matching modules. For each gene and each module, their normalized mutual information (MI) was calculated as MI = $a\;\;\log_{2}\frac{a}{\left(a+b)(a+c\right)}+d\;\;\log_{2}\frac{d}{\left(c+d)(b+d\right)}$, where *a*, *b*, *c*, and *d* are the added relative weights of (*a*) the genomes from the module that contain the gene, (*b*) the genomes that contain the gene but do not belong to the module, (*c*) the genomes from the module that lack the gene, and (*d*) the genomes that neither belong to the module nor contain the gene. Note that, compared to the traditional formulation of the mutual information, we did not take into account the terms associated with *b* and *c*. In doing so, we excluded the contributions from genes and modules with complementary patterns. The mutual information was subsequently normalized by the joint entropy: $H=-\sum_{i\;\in \;\{a,b,c,d\}}i\;\;\log_{2}i$. For each gene, we selected the two highest values of the normalized mutual information (those that corresponded to the best and second best matching modules) and used the graphical representation in Fig. 6B to set the “signature” thresholds (>0.6 for the best match, <0.02 for the second best match).

### Bacteriophage lifestyles.

Information on the temperate or virulent lifestyle of 171 tailed bacteriophages was collected from (103) (data are available at ACLAME website, http://aclame.ulb.ac.be/Classification/Phages/life_style.html). Of these phages, 57 were virulent and 114 were temperate. The number of phages with available data is much smaller than the total number of phages in our network; therefore, we discarded those modules for which there were no data for at least 10% of its members. For the remaining modules, a binomial exact test was applied to estimate the significance of the lifestyle-module association under the null hypothesis that module composition is random with respect to phage lifestyle.

## SUPPLEMENTAL MATERIAL

Table S1  List of core genes sorted by normalized abundance. Table S1, XLSX file, 0.1 MB

Table S2  List of genes shared by multiple modules (connector genes). Table S2, XLSX file, 0.05 MB

Table S3  List of genomes and module assignations. Table S3, XLSX file, 0.02 MB
